# Temporomandibular disorders, pain in the neck and shoulder area, and headache among musicians

**DOI:** 10.1111/joor.12886

**Published:** 2019-09-24

**Authors:** Maurits K. A. van Selms, Jetske W. Wiegers, Hedwig A. van der Meer, Jari Ahlberg, Frank Lobbezoo, Corine M. Visscher

**Affiliations:** ^1^ Department of Orofacial Pain and Dysfunction Academic Centre for Dentistry Amsterdam (ACTA) University of Amsterdam and Vrije Universiteit Amsterdam Amsterdam The Netherlands; ^2^ Department of Oral and Maxillofacial Diseases University of Helsinki Helsinki Finland

**Keywords:** headache, musician, neck‐shoulder pain, temporomandibular disorders, TMD pain, TMJ sounds

## Abstract

**Background:**

Uncertainties still exist about the role of playing musical instruments on the report of musculoskeletal complaints and headache.

**Objectives:**

To evaluate the prevalence of and risk indicators for symptoms of temporomandibular disorders, pain in the neck or shoulder, and headache among musicians.

**Methods:**

A questionnaire was distributed among 50 Dutch music ensembles.

**Results:**

The questionnaire was completed by 1470 musicians (response rate 77.0%). Of these, 371 musicians were categorised as woodwind players, 300 as brass players, 276 as upper strings players, 306 as vocalists and 208 as controls; nine musicians had not noted their main instrument. The mean age was 41.6 years (standard deviation [SD] 17.2), and 46.5% were male. Irrespective of instrumentalist group, 18.3% of the musicians reported TMD pain, 52.5% reported pain in the neck and shoulder area, and 42.5% reported headache. Of the functional complaints, 18.3% of the musicians reported TMJ sounds, whereas a jaw lock or catch on opening or on closing was reported by 7.1% and 2.4%, respectively. TMD pain was associated with playing a woodwind instrument, whereas pain in the neck and shoulder was associated with playing the violin or viola. For each complaint, oral behaviours were found as risk indicator, supplemented by specific risk indicators for the various complaints.

**Conclusions:**

The current finding that pain‐related symptoms varied widely between instrumentalist groups seems to reflect the impact of different instrument playing techniques. Playing a musical instrument appears not the primary aetiologic factor in precipitating a functional temporomandibular joint problem.

## 1 INTRODUCTION

Due to the specific body postures and loading of the muscles, tendons and joints that are involved in playing musical instruments, musicians often suffer from playing‐related musculoskeletal disorders (PRMDs).^1^, ^2^ Partly because of the fact that there is still no strict definition for PRMDs,^3^ a wide range of prevalence rates has been reported in the literature on performing arts medicine.^4^ Monotonous movements (viz., static and repetitive muscle work) and long training periods can affect the musculoskeletal structures of musicians, especially in the areas where the greatest muscular exertion occurs.^5^ In addition, performance anxiety and high levels of stress can cause or exacerbate many serious health problems among musicians, including PRMDs.^6^

The most frequently affected areas of PRMDs among musicians are the neck and shoulder.^7^ It has frequently been suggested that playing a musical instrument that loads the masticatory system creates an overload of that system, causes complaints in the muscles of mastication or the temporomandibular joints (TMJs).^8^, ^9^ These complaints may indicate the presence of temporomandibular disorders (TMDs) that are characterised by pain during function in the masticatory muscles, the pre‐auricular area and/or the TMJ; limited and/or deviated mandibular movements; and TMJ sounds (ie clicking and/or crepitus) during function.^10^ However, partly due to the low methodological quality and a large heterogeneity of the available studies, the available evidence pertaining to the work‐related part of this assumption is still limited and inconsistent.^11^ Differences in loading of the orofacial structures that are required for playing the various types of musical instruments are not always reflected in different TMD prevalence rates. For example, of the various groups of wind instruments, the metal brass instrumentalists apply the greatest forces on the perioral structures when performing the embouchure mechanism,^12^ whereas at the same time, brass instrumentalists show the lowest occurrence of musculoskeletal complaints.^7^

Various studies have indicated that pain complaints in the upper part of the body, such as neck/shoulder pain and pain‐related TMDs, are associated with reports of headache.^13^, ^14^, ^15^ Convergence of nociceptive inputs has been suggested to provide a neuro‐anatomical basis for the presence of these pains.^16^ At the same time, headache can be provoked by sustained masticatory muscle contraction, for example induced by tooth clenching.^17^, ^18^ Besides, during a musical performance, anxiety and various sources of psychological stress can be highly prevalent among musicians,^19^, ^20^ which are risk factors for headache as well.^21^ Given the uncertainties that still exist about the role of playing musical instruments on musculoskeletal complaints, combined with the fact that so far only little research has been devoted to headache among musicians, the aim of this questionnaire study was to evaluate the prevalence of and risk indicators for symptoms of temporomandibular disorders, pain in the neck and shoulder area, and headache in five groups of musicians. It was hypothesised that, for each of these musculoskeletal symptoms, differences in prevalence between the musical instrument groups would be reflected by differences in overloading of the areas where the greatest muscular exertion occurs.

## 2 MATERIAL AND METHODS

### 2.1 Data collection

This study was conducted among musicians of music ensembles (symphony orchestras, chamber music ensembles, brass bands, fanfares and choirs) from the Netherlands. In total, 90 music ensembles (including 15 choirs) were contacted by e‐mail or telephone between December 2013 and June 2016 and invited to participate in this study. In case permission for a visit at a rehearsal of the ensemble was granted (n = 50), the musicians were informed about the aim of the study and the procedure (viz., that they had to fill in a paper questionnaire). After that, all musicians who were present during the rehearsal were invited to participate, and they received an information letter with details about the study and the questionnaire. The questionnaires were anonymous and could be completed in under 10 minutes. This study was considered by the Medical Ethics Review Committee (METc) of the Vrije Universiteit (VU) Medical Center not to fall under the provisions of the Medical Research Involving Human Subjects Act, and medical ethical approval was granted. Musicians younger than 18 years were excluded from the database.

### 2.2 Outcome variables

In order to screen for musculoskeletal complaints in the masticatory system, the Dutch version of the “Symptom Questionnaire” (SQ) of the Diagnostic Criteria for Temporomandibular Disorders (DC/TMD)^22^ was implemented in the study questionnaire. The SQ solicits information for the most common types of TMDs (viz., TMD pain and TMJ sounds), as well as for intra‐articular forms of TMDs that are expressed by a functional limitation of the jaw. The questions that focused on headache and pain located in the neck and/or shoulder were a modified version (ie with a similar construct) of the SQ question used to assess the presence of TMD pain see below.

- TMD pain: “In the last 30 days, have you had pain in your jaw, temple, in the ear, or in front of the ear on either side?” (no, yes).
- Pain located in the neck or shoulder: “In the last 30 days, have you had any pain in the neck and/or shoulder?” (no, yes).
- Headache: “In the last 30 days, have you had any headache?” (no, yes).
- TMJ sounds: “In the last 30 days, have you had any jaw joint noise(s) when you moved or used your jaw?” (no, yes).
- Jaw lock or catch applicable to disc displacement without reduction with and without limited mouth opening: “In the last 30 days, have you had a jaw your lock or catch, even for a moment, so that it would not open all the way?” (no, yes).
- Jaw lock or catch, applicable to subluxation of the TMJ: “In the last 30 days, when you opened your mouth wide, did your jaw lock or catch even for a moment such that you could not close it from this wide open position?” (no, yes).

### 2.3 Independent variables

Besides asking for age and gender, all musicians were asked to fill in their main instrument; vocalists had to note “singing.” In addition, they were asked for their level of professionalism (amateur, semi‐professional or professional). The questionnaire also included questions concerning the number of years already spent to play the main instrument, and the average number of hours per day devoted to practise during the last 30 days.

An indication of daily stress was obtained by the question “How much stress did you experience in daily life during the last 30 days?” (NRS 0‐10).^23^ Similar questions were applied to inquire for stress during a rehearsal and to inquire for stress during a performance (leaving the possibility to mark “not applicable”). An indication of depression was assessed by asking the following two questions: “Have you been consistently depressed or down, most of the day, nearly every day, for the last 30 days?”, and “In the past 30 days, have you been much less interested in most things or much less able to enjoy the things you used to enjoy most of the time?” (no, yes).^24^ Both questions are included in the Mini International Neuropsychiatric Interview (MINI), which is a screening test to identify the possible presence of depression.^25^

An impression of (potentially adverse) oral behaviours was assessed using the Oral Parafunctions Questionnaire.^26^ For this study, the items belonging to the BRUX scale (for bruxism activities) and the BITE scale (eg chewing gum, biting nails) were used. By means of the lead‐in question “How often did you do the following activities, based on the last 30 days?”, the respondents rated each of the following oral behaviours: grinding during the night; grinding during the day; clenching during the night; clenching during the day; nail biting; biting on pens; and chewing gum, using a 5‐point Likert scale (viz., never, rarely, sometimes, often, always). The mean score of these seven behaviours (between 0 and 4) was used as indication for the total amount of oral behaviours.

Draft versions of the questionnaire were discussed with colleagues and several musicians in order to ensure that that the questions were unambiguous and focused on the research questions. Suggestions for improvement were integrated in the final version of the questionnaire.

### 2.4 Data analysis

First, the group of instrumentalists was divided into five categories: (a) woodwind (clarinet, saxophone, oboe, flute, etc), (b) brass (trumpet, trombone, euphonium, etc), (c) upper strings (violins and viola's), (d) vocalists and (e) other instrumentalists (cello, guitar, percussion, keyboards, etc). As it can be argued that musicians playing an instrument from the last category apply less pressure on their masticatory system compared with the other groups, this group served as control group (coded “0”). The level of professionalism was assessed by dividing the sample into two groups: amateurs vs (semi) professionals. The prevalence rates of the outcome variables and the characteristics of the independent variables were summarised for the different instrumentalist categories. Descriptive statistics also included a bar chart depicting the proportion of self‐reported symptoms of temporomandibular disorders, pain in the neck or shoulder, and headache in relation to musician group and gender (Appendix S1). To evaluate the relations of the reported symptoms to instrument category, as well as with the other independent variables, logistic regression analyses were used. First, the unadjusted associations with gender, age, type of musician, length of playing experience, hours of daily practice, level of professionalism, amount of daily stress, amount of stress during a rehearsal, amount of stress during a performance, being depressed or down, loss of interest or less joy, and number of adverse oral behaviours were assessed. All independent variables that showed at least a weak association with the outcome variable (*P*‐value <.10) were incorporated into a multiple regression model. Subsequently, in a step‐by‐step approach, the independent variable with the weakest association with the outcome variable was removed from the model, until all independent variables in the final model showed a *P*‐value <.05. To assure adequate statistical power, at least 10 participants were required for each independent variable.^27^ Besides looking at the total number of observations per independent variable, also the number of "events" was taken into account. For logistic regression, the number of "events" is the number of cases in the least‐frequent of the two outcome classes (eg pain vs no pain). For example, a particular study may have many participants, but too few persons who report pain for a valid analysis. Since the validity of the logistic regression model may be affected when the number of events per variable (EPV) is less than ten, no analysis was performed in case EPV < 10.^28^ Analyses were conducted using the IBM SPSS Statistics 25 software package (IBM Corp).

## 3 RESULTS

Based on the information that was provided to the students who performed the data acquisition, the 50 musical ensembles consisted of 1910 eligible musicians. Since not all of them were present at the time the questionnaire was handed over, the sample consisted of 1470 musicians who had completed the questionnaire (response rate 77.0%). Of these, 371 musicians were categorised as woodwind players, 300 as brass players, 276 as upper strings players, 306 as vocalists and 208 as controls; nine musicians had not noted their main instrument. The mean age of all participants was 41.6 years (standard deviation [SD] 17.2). Moreover, 46.5% of the participants were male. Descriptive statistics of all variables included in this study, depicted for each instrumentalist category, are shown in Table 1. The highest prevalence of TMD pain was reported by vocalists (viz., 21.9%), whereas self‐reported pain in the neck and shoulder area was most prevalent among the upper string players (69.2%). Headache had the highest occurrence among vocalists (45.5%) and the upper string players (45.4%). Of the functional complaints related to TMDs, self‐reported TMJ sounds were most prevalent among the upper string players (21.0%), and both a jaw lock or catch on opening and jaw lock or catch on closing were most reported by vocalists (10.5% and 3.7%, respectively).

**Table 1:** Characteristics of the total sample stratified by instrumentalist category. Continuous variables are presented as mean value (standard deviation); dichotomous variables are presented as absolute numbers (percentage)

	Overall	Woodwind	Brass	Upper strings	Vocalists	Controls
	N = 1461	N = 371	N = 300	N = 276	N = 306	N = 208
Independent variables						
Age, y, mean (SD)	41.6 (17.2)	43.0 (16.3)	43.1 (16.0)	41.6 (17.5)	37.5 (17.7)	42.6 (18.0)
Gender, female, n (%)	780 (53.5)	225 (61.5)	78 (26.2)	195 (72.0)	195 (63.9)	85 (40.9)
Professionalism, (semi)professional, n (%)	460 (31.5)	97 (26.1)	75 (25.2)	80 (29.3)	133 (43.5)	73 (35.6)
Playing experience, y, mean (SD)	24.8 (14.7)	25.9 (13.5)	25.9 (13.9)	29.4 (15.2)	18.5 (13.9)	24.1 (15.2)
Playing intensity per day, h, mean (SD)	1.8 (1.8)	1.5 (1.6)	1.4 (1.6)	1.9 (2.0)	2.1 (2.0)	2.0 (2.0)
Stress daily life, mean (SD)	4.0 (2.8)	3.6 (2.8)	3.6 (2.8)	4.3 (2.7)	4.6 (2.6)	3.6 (3.0)
Stress rehearsal, mean (SD)	2.1 (2.3)	1.8 (2.1)	2.0 (2.2)	2.6 (2.4)	2.6 (2.5)	1.8 (2.1)
Stress performance, mean (SD)	3.0 (2.7)	2.7 (2.5)	2.9 (2.6)	3.7 (2.7)	3.5 (2.9)	2.5 (2.3)
Depressed or down, yes, n (%)	81 (5.6)	13 (3.5)	12 (4.0)	12 (4.4)	34 (11.2)	10 (4.8)
Loss of interest, yes, n (%)	187 (12.9)	39 (10.7)	31 (10.4)	37 (13.7)	59 (19.5)	20 (9.7)
Oral behaviours, mean (SD)	0.4 (0.5)	0.4 (0.4)	0.4 (0.5)	0.4 (0.5)	0.5 (0.5)	0.4 (0.5)
Outcome variables						
TMD pain, n (%)	268 (18.3)	74 (20.1)	46 (15.4)	54 (19.7)	67 (21.9)	25 (12.0)
Pain in neck and shoulder area, n (%)	762 (52.5)	195 (53.4)	123 (41.6)	189 (69.2)	158 (51.8)	96 (46.6)
Headache, n (%)	618 (42.5)	150 (41.0)	117 (39.1)	124 (45.4)	138 (45.5)	88 (42.9)
TMJ sounds, n (%)	266 (18.3)	71 (19.1)	46 (15.4)	57 (21.0)	60 (20.0)	31 (15.2)
Jaw lock or catch on opening, n (%)	103 (7.1)	27 (7.3)	13 (4.3)	20 (7.3)	32 (10.5)	11 (5.4)
Jaw lock or catch on closing, n (%)	34 (2.4)	10 (2.7)	4 (1.4)	3 (1.1)	11 (3.7)	6 (2.9)

Note

The control group consisted of musicians for whom loading of the masticatory system is not required for the musical performance

For each instrumentalist category and stratified by gender, the data of the three pain conditions (viz., TMD pain, pain in the neck and shoulder area, and headache) and of the three types of functional complaints (viz., TMJ sounds, jaw lock or catch on opening, and jaw lock or catch on closing) are depicted in S1. Based on this figure, there seems to be a trend that female musicians reported pain complaints more frequently than male musicians (see below).

In Tables 2, 3, 4, 5, 6, the outcomes of the single and multiple logistic regression analyses with respect to the report of the various outcome variables among musicians are presented. Regarding the report of TMD pain by musicians, the multiple regression analyses indicated that being a woodwind player, having a younger age, showing loss of interest and having adverse oral behaviours were associated with higher odds for having TMD pain (Table 2). Being an upper string instrument player, female and younger, having higher playing intensity, showing loss of interest and having more adverse oral behaviours were the best predictors of pain in the neck and shoulder area according to the final model in Table 3. As can be seen in Table 4, there was no association between the type of musician and the self‐report of headache. Instead, female gender, younger age, a higher levels of stress during daily life, having less interest in things, and a higher score for oral behaviours were positively associated with headache in the multiple regression model. Regarding the report of TMJ sounds, performance stress and oral behaviours were retained in the final model (Table 5). Finally, Table 6 presents the results of the single and multiple logistic regression analyses with respect to the report of jaw lock or catch on opening among musicians. After correction for the influence of all variables that were initially included in the final model (viz., type of musician, age, playing experience, stress daily life/ rehearsal/ performance, feeling depressed or down, and oral behaviours), it appeared that a jaw lock or catch on opening was associated with younger age and more adverse oral behaviours. Statistics on the report of jaw lock or catch on closing were not executed as the proportion of positive cases in the upper strings category was only three (see Table 1), which was lower than the required minimum of ten.

**Table 2:** Single and multiple logistic regression models of variables associated with TMD pain among musicians (n = 1,461). Associations are expressed as odds ratio (OR) and 95% confidence interval (CI). For each removed independent variable, the P‐to‐Exit is reported

Outcome variable: TMD pain					
Independent variable	Single regression models		P‐to‐Exit	Multiple regression model	
	*P* value	OR (95% CI)		*P* value	OR (95% CI)
Type of musician					
Control group		Reference			Reference
Woodwind	0.015	1.84 (1.13‐3.00)		0.010	2.20 (1.21‐4.00)
Brass	0.277	1.34 (0.79‐2.25)		0.283	1.42 (0.75‐2.70)
Upper strings	0.025	1.78 (1.08‐3.00)		0.226	1.48 (0.78‐2.79)
Vocalists	0.005	2.05 (1.25‐3.38)		0.126	1.62 (0.87‐2.99)
Gender					
Male		Reference			
Female	0.001	1.60 (1.22‐2.10)	0.130	–	–
Age (y)	<0.001	0.97 (0.96‐0.98)		<0.001	0.98 (0.97‐0.99)
Playing experience (y)	<0.001	0.97 (0.96‐0.98)	0.258		
Playing intensity per day (h)	0.033	1.08 (1.01‐1.16)	0.168	–	–
Professionalism					
Amateur		Reference			
(Semi) professional	0.006	1.47 (1.11‐1.93)	0.891	–	–
Stress daily life (0‐10)	<0.001	1.10 (1.04‐1.15)	0.782	–	–
Stress rehearsal (0‐10)	<0.001	1.11 (1.05‐1.17)	0.537	–	–
Stress performance (0‐10)	0.164	1.04 (0.98‐1.10)			
Depressed or down					
No		Reference			
Yes	0.008	1.97 (1.20‐3.23)	0.938	–	–
Loss of interest					
No		Reference			Reference
Yes	<0.001	2.15 (1.52‐3.04)		0.038	1.62 (1.03‐2.54)
Oral behaviours (0‐4)	<0.001	3.28 (2.43‐4.43)		<0.001	2.64 (1.91‐3.65)

**Table 3:** Single and multiple logistic regression models of variables associated with pain in the neck and shoulder area among musicians (n = 1,461). Associations are expressed as odds ratio (OR) and 95% confidence interval (CI). For each removed independent variable, the P‐to‐Exit is reported

Outcome variable: pain in neck and shoulder area					
Independent variable	Single regression models		P‐to‐Exit	Multiple regression model	
	*P* value	OR (95% CI)		*P* value	OR (95% CI)
Type of musician					
Control group		Reference			Reference
Woodwind	0.118	1.31 (0.93‐1.85)		0.545	1.15 (0.74‐1.78)
Brass	0.262	0.82 (0.60‐1.17)		0.351	0.81 (0.51‐1.27)
Upper strings	<0.001	2.58 (1.77‐3.75)		0.018	1.78 (1.10‐2.86)
Vocalists	0.249	1.23 (0.86‐1.76)		0.133	0.70 (0.45‐1.11)
Gender					
Male		Reference			
Female	<0.001	2.38 (1.93‐2.94)		<0.001	1.92 (1.44‐2.56)
Age (y)	<0.001	0.97 (0.97‐0.98)		<0.001	0.98 (0.97‐0.98)
Playing experience (y)	<0.001	0.98 (0.98‐0.99)	0.943	–	–
Playing intensity per day (h)	0.006	1.09 (1.03‐1.16)		0.012	1.10 (1.02‐1.18)
Professionalism					
Amateur		Reference			
(Semi) professional	0.002	1.43 (1.14‐1.78)	0.218	–	–
Stress daily life (0‐10)	<0.001	1.08 (1.04‐1.12)	0.934	–	–
Stress rehearsal (0‐10)	0.001	1.08 (1.03‐1.14)	0.990	–	–
Stress performance (0‐10)	0.011	1.06 (1.01‐1.11)	0.342	–	–
Depressed or down					
No		Reference			
Yes	0.017	1.77 (1.11‐2.84)	0.617	–	–
Loss of interest					
No		Reference			Reference
Yes	<0.001	2.35 (1.68‐3.27)		0.004	1.89 (1.23‐2.90)
Oral behaviours (0‐4)	<0.001	2.18 (1.65‐2.88)		0.033	1.39 (1.03‐1.88)

**Table 4:** Single and multiple logistic regression models of variables associated with headache among musicians (n = 1,461). Associations are expressed as odds ratio (OR) and 95% confidence interval (CI). For each removed independent variable, the P‐to‐Exit is reported

Outcome variable: headache					
Independent variable	Single regression models		P‐to‐Exit	Multiple regression model	
	*P* value	OR (95% CI)		*P* value	OR (95% CI)
Type of musician					
Control group		Reference			
Woodwind	0.651	0.92 (0.65‐1.31)			
Brass	0.394	0.86 (0.60‐1.23)			
Upper strings	0.587	1.11 (0.77‐1.59)			
Vocalists	0.560	1.11 (0.78‐1.60)			
Gender					
Male		Reference			
Female	<0.001	2.31 (1.86‐2.86)		<0.001	1.81 (1.39‐2.37)
Age (y)	<0.001	0.96 (0.95‐0.97)		<0.001	0.97 (0.96‐0.98)
Playing experience (y)	<0.001	0.97 (0.96‐0.97)	0.455	–	–
Playing intensity per day (h)	0.233	1.04 (0.98‐1.10)			
Professionalism					
Amateur		Reference			
(Semi) professional	0.351	1.11 (0.89‐1.39)			
Stress daily life (0‐10)	<0.001	1.18 (1.14‐1.23)		0.005	1.08 (1.02‐1.13)
Stress rehearsal (0‐10)	<0.001	1.11 (1.05‐1.16)	0.137	–	–
Stress performance (0‐10)	0.002	1.07 (1.03‐1.12)	0.137	–	–
Depressed or down					
No		Reference			
Yes	0.049	1.58 (1.00‐2.49)	0.743	–	–
Loss of interest					
No		Reference			Reference
Yes	<0.001	2.23 (1.63‐3.06)		0.001	2.05 (1.33‐3.14)
Oral behaviours (0‐4)	<0.001	2.86 (2.16‐3.79)		<0.001	1.79 (1.32‐2.42)

**Table 5:** Single and multiple logistic regression models of variables associated with TMJ sounds among musicians (n = 1,461). Associations are expressed as odds ratio (OR) and 95% confidence interval (CI). For each removed independent variable, the P‐to‐Exit is reported

Outcome variable: TMJ sounds					
Independent variable	Single regression models		P‐to‐Exit	Multiple regression model	
	*P* value	OR (95% CI)		*P* value	OR (95% CI)
Type of musician					
Control group		Reference			
Woodwind	0.237	1.32 (0.83‐2.10)			
Brass	0.954	1.02 (0.62‐1.66)			
Upper strings	0.106	1.49 (0.92‐2.41)			
Vocalists	0.170	1.40 (0.87‐2.25)			
Gender					
Male		Reference			
Female	0.039	1.33 (1.01‐1.74)	0.093	–	–
Age (y)	<0.001	0.98 (0.97‐0.98)	0.246		
Playing experience (y)	<0.001	0.98 (0.97‐0.99)	0.730	–	–
Playing intensity per day (h)	0.070	1.07 (1.00‐1.15)	0.079	–	–
Professionalism					
Amateur		Reference			
(Semi) professional	0.015	1.41 (1.07‐1.87)	0.700	–	–
Stress daily life (0‐10)	0.002	1.08 (1.03‐1.13)	0.138	–	–
Stress rehearsal (0‐10)	<0.001	1.13 (1.07‐1.20)	0.493	–	–
Stress performance (0‐10)	<0.001	1.10 (1.05‐1.16)		0.009	1.09 (1.02‐1.16)
Depressed or down					
No		Reference			
Yes	0.204	1.41 (0.83‐2.41)			
Loss of interest					
No		Reference			
Yes	0.162	1.31 (0.90‐1.90)			
Oral behaviours (0‐4)	<0.001	2.43 (1.81‐3.27)		<0.001	2.21 (1.60‐3.07)

**Table 6:** Single and multiple logistic regression models of variables associated with jaw lock or catch on opening among musicians (n = 1,461). Associations are expressed as odds ratio (OR) and 95% confidence interval (CI). For each removed independent variable, the P‐to‐Exit is reported

Outcome variable: jaw lock opening					
Independent variable	Single regression models		P‐to‐Exit	Multiple regression model	
	*P* value	OR (95% CI)		*P* value	OR (95% CI)
Type of musician					
Control group		Reference	0.642	–	–
Woodwind	0.378	1.38 (0.67‐2.85)			
Brass	0.599	0.80 (0.35‐1.83)			
Upper strings	0.391	1.39 (0.65‐2.98)			
Vocalists	0.041	2.10 (1.03‐4.27)			
Gender					
Male		Reference			
Female	0.225	1.29 (0.86‐1.93)			
Age (y)	<0.001	0.95 (0.94‐0.97)		<0.001	0.96 (0.94‐0.98)
Playing experience (y)	<0.001	0.94 (0.93‐0.96)	0.151	‐	–
Playing intensity per day (h)	0.729	0.98 (0.87‐1.10)			
Professionalism					
Amateur		Reference			
(Semi) professional	0.643	0.90 (0.58‐1.40)			
Stress daily life (0‐10)	0.007	1.11 (1.03‐1.19)	0.492	–	–
Stress rehearsal (0‐10)	0.095	1.08 (0.99‐1.17)	0.689	–	–
Stress performance (0‐10)	0.036	1.09 (1.01‐1.19)	0.133	–	–
Depressed or down					
No		Reference			
Yes	0.052	1.99 (0.99‐4.00)	0.821	–	–
Loss of interest					
No		Reference			
Yes	0.138	1.50 (0.88‐2.56)			
Oral behaviours (0‐4)	<0.001	2.27 (1.54‐3.35)		0.024	1.64 (1.07‐2.51)

## 4 DISCUSSION

The first aim of this study was to determine the prevalence of self‐reported temporomandibular disorders, pain in the neck or shoulder, and of headache in musicians. The results showed that 18.3% of the musicians reported TMD pain, 52.5% reported pain in the neck and shoulder area, and 42.5% reported headache. Of the functional complaints, 18.3% of the musicians reported TMJ sounds, a jaw lock or catch on opening was reported by 7.1%, whereas only 2.4% of the musicians reported a jaw lock or catch on closing. The second aim was to evaluate the risk indicators that are associated with the presence of these complaints. For each complaint, oral behaviours were found as risk indicator, supplemented by specific risk indicators for the various complaints (see below).

Previously reported prevalence rates on TMD pain among musicians are comparable to that observed in the present study (viz., 18.3%). First, a recent study mentioned that 21.1% of the 739 musicians reported pain around the cheeks, temple, or jaw.^29^ Others found that 23% 9 and 28.9%^30^ of the orchestra players reported TMD pain in the past month. In all three studies, however, TMD pain was not found to be associated with a specific instrumentalist group. This lack of evidence for differences in prevalence between instrumentalist groups might be related to the relatively low number of participants in some of their groups. In the current study, playing instruments of the woodwind category appeared to be associated with self‐reported TMD pain. This corroborates with the study of Yasuda et al (2016), who found that the prevalence of a mixture of symptoms of TMDs among 184 junior high school students playing wind instruments was higher than in the 26 students who played other (non‐wind) instruments.^31^ The authors ascribed this finding to the possibility that playing wind instruments imposes a strain on the jaw muscles. This is, however, contrary to the results of an experimental study, showing that the contractive load to jaw‐closing muscles when playing a wind instrument actually appeared to be very small.^32^ As playing a wind instrument for 90 minutes did not obviously induce fatigue of jaw‐closing muscles, the authors concluded that there seems to be little possibility of wind instrument playing being a causal factor of TMDs. Of course, it should be reminded that the potential adverse effects of playing a wind instrument for many hours per day, or for many years, can never be replicated in an experimental study. There might, however, also be another explanation for the current finding that woodwind players reported more TMD pain. Since playing wind instruments involves the arrangements of the facial muscles and lips to produce a sound, this type of instrumentalists might be more aware of complaints in the orofacial area as compared to musicians using other anatomical structures (eg arm, hand). More studies are needed in order to clarify the mechanisms involved in the report of TMD pain in woodwind players.

The observed high occurrence of self‐reported pain in the neck and shoulder area among upper string musicians (viz., 69.2%) is in accordance with previous studies.^33^, ^34^ Playing the violin and the viola requires a prolonged external shoulder rotation, flexion of the head, arm elevation and constant supination of the left forearm, which can cause overuse injuries predominantly in the left upper limb.^35^ The multiple regression model also revealed a significant association between playing intensity and the report of pain in the neck and/or shoulders. This coincides with knowledge on the field of work physiology, namely that the length of daily working hours and perceived physical workload are risk factors for the development of playing‐related musculoskeletal disorders (PRMDs) among musicians.^1^, ^6^ The final regression model further indicated that female gender was highly associated with this pain. This combined with the finding that female gender was associated with three other outcome variables according to the unadjusted single regression analyses, confirms the conclusion of Paarup et al that a pronounced gender difference may exist, with a higher prevalence of musculoskeletal symptoms among female musicians than male musicians.^36^ Oral behaviours were also found to be associated with pain in the neck and shoulder area, which at first sight seems unexpected. However, it has been found that the report of oral behaviours is confounded by other variables, as oral behaviours appear to be associated with stress, headache and TMD pain, while at the same time TMD pain and stress being associated with headache and neck complaints.^13^, ^37^, ^38^ In line with this is the finding that stress, neck/shoulder pain and headaches are all associated with each other.^39^ The exact nature of these associations is unknown, but it is thought that peripheral and central sensitisation play an important role in TMD pain, headaches and neck/shoulder complaints, especially when stress is involved.^40^ Future research should look into this association matrix of variables to establish the role of oral behaviours in musicians with different types of pain complaints.

The current finding that headache was reported by 41.0% (woodwind players) to 45.5% (vocalists) of the musicians is comparable with the observation that current headache occurs in 53% of adults as reported in a systematic literature review on European studies.^41^ Further, the finding that self‐reported headache was not associated with the type of musician corroborates with the outcome of a recent study among 408 professional orchestra musicians, investigating six groups of musicians.^34^ On the other hand, the suggestion that being a choral singer can be considered a protective factor for the occurrence of headaches could not be replicated.^42^ Of course, it should be reminded that the current study did not differentiate between the different types of headache, such as migraine and tension‐type headache. Future research might explore if an association exists between the different types of headache and musical instrument induced masticatory loading. In the current study, it appeared that female gender, younger age, daily stress, having less interest in things, and oral behaviours were positively associated with the report of headache. These findings are not surprising, because headache is usually reported more often by women than men,^41^ and headache sufferers score higher on perceived stress than control subjects.^43^ Depression and painful symptoms commonly occur together, because they share neurobiological pathways and neurotransmitters.^44^ It has also been suggested that various types of headache are associated with mechanical loading of the masticatory muscles.^17^, ^38^ This association, however, is not well understood.

Literature on TMJ sounds among musicians is relatively scarce and yields ambiguous outcomes.^11^ The results of the present study indicate that TMJ sounds were present in about 15%‐20% of the musicians (Table 1), which is comparable to the prevalence rate found in the general population (viz., 23.7%).^45^ In the current study, the presence of self‐reported TMJ sounds was not associated with the type of musician. This is in line with Heikkilä et al, who found that the occurrence of TMJ sounds did not vary between the instrumentalist groups.^46^ They found that TMJ clicking sounds were present in 27% of the musicians. On the other hand, Jang et al reported a higher occurrence of clicking or popping sounds among musicians (viz., 45.7%), with the highest prevalence in woodwind and brass instrumentalists.^29^ The association between stress during a performance and TMJ sounds is difficult to explain, because the presence of such sounds usually simply reflects natural variation.^47^ Nevertheless, it has been suggested that psychological factors may be indirectly associated with TMJ sounds, involving stress‐induced oral behaviours.^48^ The applied heavy forces would lead to high compressive forces within the TMJ and thus to more joint sounds. As links between psychological factors and TMJ sounds have received little attention, future studies are needed to more fully explore the underlying mechanisms.

To the best of our knowledge, only few studies have inquired for limitations of jaw opening among musicians, which makes it difficult to compare the present findings. According to our study, a jaw lock or catch on opening was reported by 7.1% of the musicians. In a study by Steinmetz et al (2014), limitation of jaw opening was reported by 0%‐18% of the six instrumentalist groups.^9^ A survey among 135 amateur wind instrumentalists suggested that 13% reported a history of jaw catching and locking.^49^ The results of the present study showed that oral habits were positively associated with the report of a jaw lock or catch on opening. This is in line with the conclusion of a study by Kalaykova et al, showing that diurnal clenching may be a risk factor for intermittent locking.^50^ The present finding that jaw lock or catch on opening was associated with being a vocalist according to the unadjusted regression model, could be related to the earlier mentioned awareness as well. As vocalists demand high physical strains of the masticatory system, they might be more aware of complaints in that same orofacial area in comparison with other musicians.

The present study has several limitations. Due to the cross‐sectional design, the observed findings merely reveal associations that require further testing in order to show cause and effect. Another drawback deals with the subjective nature. The presence of the various symptoms of temporomandibular disorders, pain in the neck or shoulder, and of headache were assessed through self‐report only. Although a clinical examination to confirm the presence of these complaints would have enhanced the validity, it would at the same time have reduced the number of participants. It should also be noted that many musicians played multiple instruments. Even though we checked if this variable was associated with the outcome variables (not significant; data not shown), a potential bias can not be ruled out. In line with this is the fact that most instrumentalist categories were not uniform with respect to the type of instrument. For example, the woodwind category consisted of a mixture of musical instruments with a large variation in size, playing position and technique.

In conclusion, 18.3% of the 1470 musicians who completed the questionnaire reported TMD pain, 52.5% reported pain in the neck and shoulder area, and 42.5% reported headache. For the functional complaints, the prevalence of self‐reported TMJ sounds was 18.3%, a jaw lock or catch on opening was reported by 7.1%, whereas only 2.4% of the musicians reported a jaw lock or catch on closing. TMD pain appeared to be associated with playing a woodwind instrument, whereas pain in the neck and shoulder area was associated with playing the violin or viola. Moreover, oral behaviours were found to be associated with all pain and functional outcome measures. The current finding that pain‐related symptoms varied widely between instrumentalist groups seems to reflect the impact of different instrument playing techniques. Combining all evidence together, playing a musical instrument seems not the primary aetiologic factor in precipitating a functional TMJ problem.

## CONFLICT OF INTEREST

None declared.
