# Supplementary figures and images for: Temporomandibular disorders, pain in the neck and shoulder area, and headache among musicians
Source: J Oral Rehabil. 2019 Sep 24;47(2):132–42. doi: 10.1111/joor.12886 (PMC7004094; doi:10.1111/joor.12886)

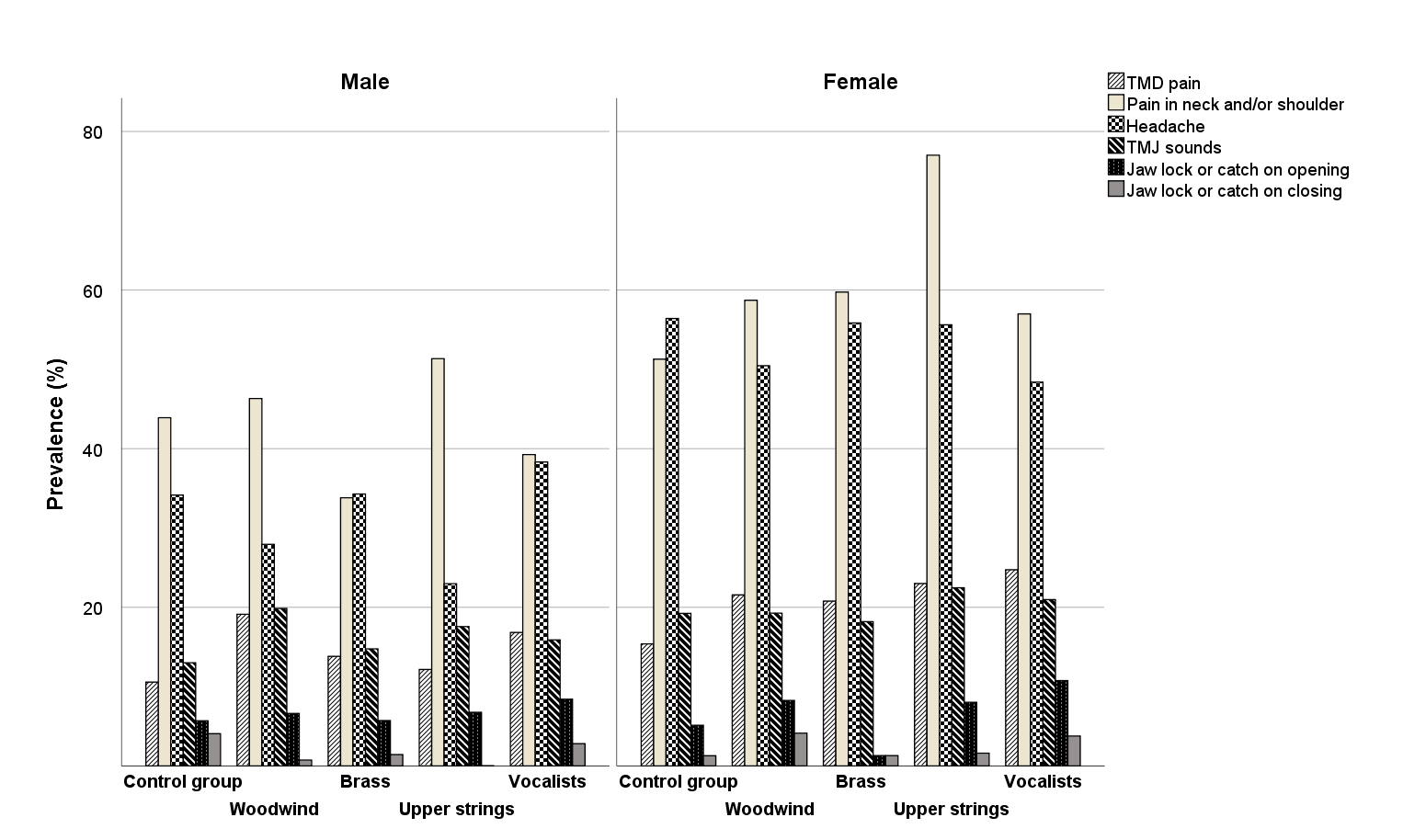

Supplement: Supplementary file 1 [file JOOR-47-132-s001.bmp]
